# Efficacy and safety of magnetic resonance-guided focused ultrasound for Parkinson’s disease: a systematic review and meta-analysis

**DOI:** 10.3389/fneur.2023.1301240

**Published:** 2023-12-07

**Authors:** Xiaona Tian, Rongrui Hu, Peicong He, Jianhong Ye

**Affiliations:** ^1^Eighth Clinical School, Guangzhou University of Chinese Medicine, Foshan, China; ^2^Endocrinology Department, Foshan Hospital of Traditional Chinese Medicine, Foshan, China

**Keywords:** MRgFUS, Parkinson’s disease, efficacy, safety, Meta-analysis

## Abstract

**Objective:**

Magnetic resonance imaging-guided focused ultrasound (MRgFUS) is a novel noninvasive treatment for drug-resistant Parkinson’s disease (PD) related tremor. This study aims to evaluate MRgFUS’s efficacy and safety in PD through a systematic review and meta-analysis, examining pre-and post-treatment MDS-UPDRSIII and/or CRST scores and associated adverse events.

**Materials and methods:**

We conducted an extensive literature search across PubMed, Embase, Web of Science, and Cochrane Library databases, screening studies based on set criteria and analyzing MDS-UPDRSIII, CRST, and adverse events pre- and post-MRgFUS treatment.

**Results:**

Out of 468 retrieved articles, 20 studies involving 258 patients, spanning 2014–2023, were included.17 studies indicated significant MDS-UPDRSIII score reductions post-MRgFUS treatment, while 3 showed significant CRST score declines. In the “on” medication state, pooled MDS-UPDRSIII scores at 1, 3, 6, and 12 months were 12.18 (95% CI: 5.83–18.52), 12.10 (95% CI: 8.22–15.97), 14.85 (95% CI: 9.28–20.41), and 20.65 (95% CI: 12.15–29.14) respectively. In the “off” state, scores were 11.45 (95% CI: −3.50-26.40), 14.71 (95% CI: 4.95–24.46), 21.52 (95% CI: 19.28–23.75), and 22.28 (95% CI: 15.26–29.30). Adverse events were typically mild and transient, with speech disturbances, ataxia, and sensory abnormalities being common post-operative neurological complications.

**Conclusion:**

MRgFUS offers an effective and relatively safe treatment option for patients with drug-resistant PD-related tremor.

**Systematic review registration:**

https://www.crd.york.ac.uk/prospero/, No. CRD42023428332.

## 1 Introduction

Parkinson’s disease (PD) is a common neurodegenerative disorder, with the risk of onset increasing with age (1). The clinical manifestations of this disease include motor-related symptoms such as bradykinesia, rigidity, and tremor, as well as non-motor symptoms including impaired olfaction, cognitive disorders, and psychiatric disturbances (2). These symptoms significantly impact the quality of life of the patients, bringing immense psychological and medical burdens to their families (3).

The primary treatment strategy for PD is usually pharmacotherapy, which includes anticholinergic agents, dopaminergic receptor agonists and levodopa (4).These medications can help alleviating patients’ symptoms and improve their quality of life. For those who do not respond well to medications or experience significant side effects, surgical treatment becomes an alternative option. Currently, Deep Brain Stimulation (DBS) is the predominant surgical approach for treating PD, particularly interventions targeting the ventral intermediate nucleus (VIM), globus pallidus internus (GPI), and subthalamic nucleus (STN) (5, 6). This is suitable for advanced PD, when oral or transdermal treatments are no longer effective (7). However, despite the adjustable advantages of DBS, it is important to note that this method is invasive and quite costly, in addition to the risks associated with device implantation and electrical stimulation, which cannot be ignored (6, 8).

.Magnetic Resonance Imaging-guided Focused Ultrasound (MRgFUS) is a non-invasive neurosurgical technique that offers minimally invasive ablation, paving a new way for the treatment of PD-related tremor (9). This technique is characterized by its non-invasiveness, lack of radiation, and the absence of a need for anesthesia (10, 11). It works by thermally ablating specific brain regions (such as VIM and GPI) during the treatment process, forming a coagulative necrotic focus (12). Compared to traditional invasive surgeries, MRgFUS significantly reduces the risks of infection and cerebral hemorrhage (13). Studies have demonstrated its safety and efficacy in treating Essential Tremor (ET) (14, 15). However the safety and efficacy of MRgFUS in treating PD related tremor and in improving other PD symptoms need to be better elucidated. Currently, there is a relatively limited literature review on the efficacy and safety of MRgFUS in the treatment of Parkinson’s disease (16, 17). In terms of relevant reports for which meta-analysis was performed, only one article exists, and that article included only two papers (18). In contrast, our study employs a single-arm meta-analysis approach that encompasses a wider range of research literature and takes into account a longer span of years to provide a more comprehensive and in-depth analysis. This will help to fill the knowledge gap in the existing literature. Therefore, our research aims to systematically review relevant literature to assess the safety and efficacy of MRgFUS in treating drug-resistant PD-related tremor. Our study results may provide a scientific basis for the clinical application of MRgFUS in the treatment of drug-resistant PD-related tremor.

## 2 Methods and analysis

### 2.1 Purpose and registration

A systematic review and meta-analysis will be performed to synthesize the evidence and assess the efficacy and safety of MRgFUS for the treatment of drug-resistant PD-related tremor. This protocol is registered in PROSPERO (No. CRD42023428332). This study followed the Preferred Reporting Items for Systematic Reviews and Meta-Analyzes (PRISMA) statement (19).

### 2.2 Information sources and search strategies

A systematic search was conducted on August 6, 2023, using PubMed, Embase, Web of science, and Cochrane Library electronic databases for the keywords (“MRgFUS” or “HIFU” or “focused ultrasound”) AND (“Parkinson disease”).

Inclusion criteria: articles must report on the efficacy and/or safety of MRgFUS treatment in patients with PD. For efficacy: quantitative or qualitative data on major symptoms such as tremor, bradykinesia, and rigidity need to be included. For safety: need to include the incidence and detailed description of complications, adverse events associated with MRgFUS therapy; the intervention in the study was the administration of MRgFUS treatment to the study cases. Exclusion criteria: studies with less than 3 patients, case reports, experimental animal studies, reviews, conference abstracts, duplicate publications, or literature with missing data.

### 2.3 Data extraction and analysis

**Figure 1:**
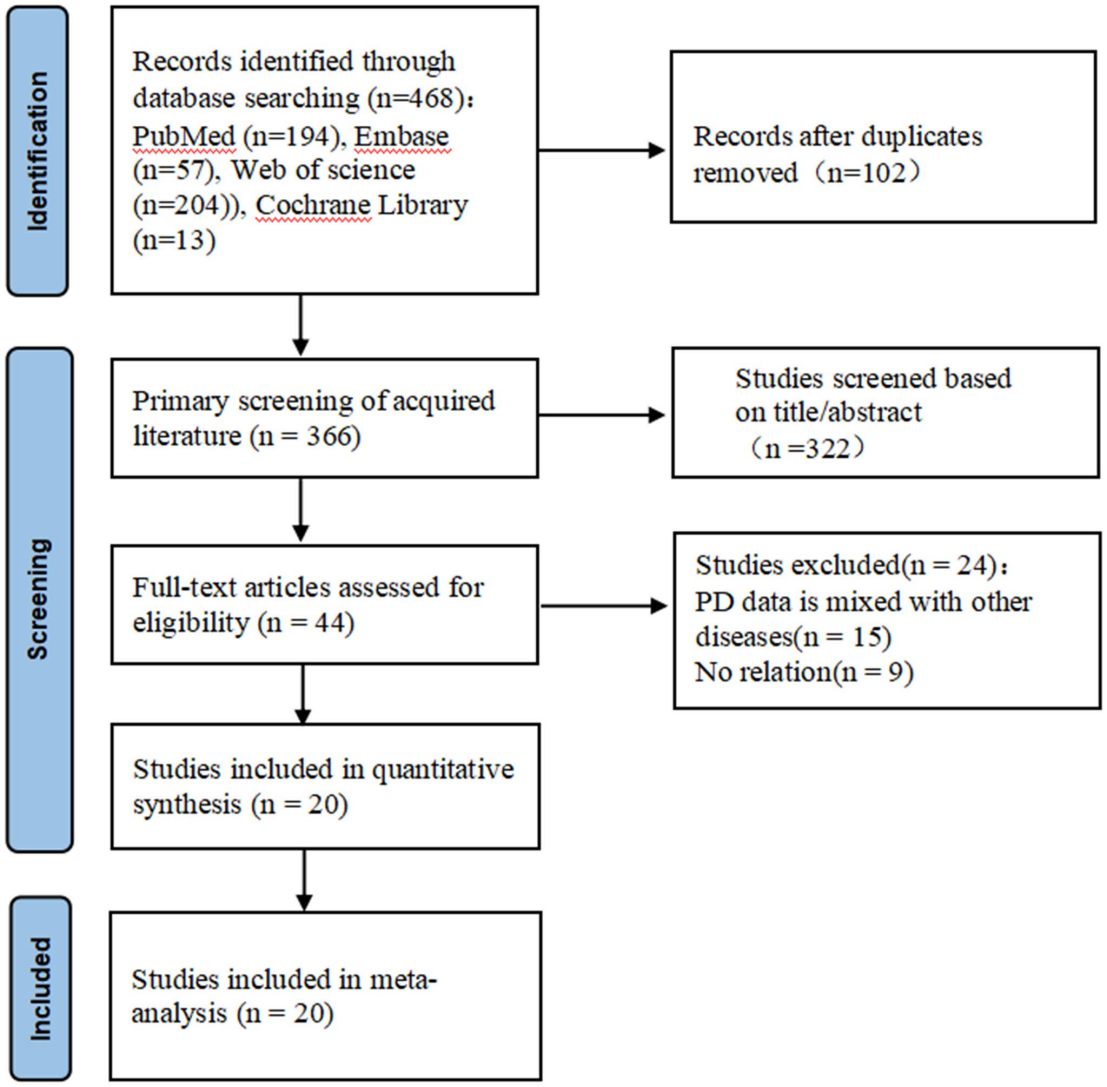
Flowchart of literature search and study selection process.

Two independent researchers undertook the literature search. After deduplicating with NoteExpress, abstracts and titles were preliminarily screened based on inclusion criteria. Relevant full-text articles were further assessed, and in cases of data overlap, only the most updated or comprehensive study was retained. Disparities between researchers were reconciled through consultation with a third expert. Subsequently, a collective in-depth analysis and data extraction from the selected studies were done, ensuring unanimous agreement on divergences. The selection process is illustrated in the PRISMA flow diagram (Figure 1). Essential details for each study, such as authorship, publication date, study design, sample demographics, PD duration, follow-up duration, efficacy metrics (including both on- and off-medication Movement Disorder Society Unified Parkinson’s Disease Rating Scale part III (MDS-UPDRSIII) scores, and/or pre- and post-treatment Clinical Rating Scale for Tremor (CRST) scores), recurrent events of tremor and postoperative adverse events, were systematically recorded. The MDS-UPDRSIII scale usually covers a comprehensive assessment of hand movements, upper extremity movements, and lower extremity movements. Increased patient scores in these areas usually reflect severe impairments in movement in patients with PD. The CRST score is primarily used to assess resting and locomotor tremor at different sites, as well as other symptoms associated with tremor. Higher MDS-UPDRSIII scores indicate more severe impairment of motor function in patients with PD, and higher CRST scores indicate more significant symptoms of resting and motor tremor in patients with PD.

### 2.4 Statistical analysis

Meta-analysis was performed using the R language (v4.2.2) meta-function package to meta-analyze the data, with measurements expressed as mean difference and standard deviation (MD ± SD), and dichotomous data expressed as proportions and 95% confidence intervals. Heterogeneity among the results of the included studies was tested with I^2^ and *p*-values, and if there was good statistical homogeneity among the studies (*p* > 0.1; *I*^2^ ≤ 50%), Meta-analysis was performed using a fixed-effects model; if there was statistical heterogeneity (*p* < 0.1; *I*^2^ > 50%), Meta-analysis could be performed using a random-effects model. The level of test for Meta was *α* = 0.05 with a statistically significant *p* < 0.05.

## 3 Results

### 3.1 Study characteristics

This meta-analysis included 20 studies published between 2014 and 2023 (5, 9, 11, 20–36), encompassing 258 patients. Among these, two were retrospective studies and 18 were prospective. Table 1 provides a detailed breakdown of the characteristics of each study. The participants were primarily middle-aged and elderly, with a majority being male. The follow-up duration varied across studies, ranging from as short as 1 month to as long as 3 years. Three studies (21, 27, 29) documented cases of bilateral pallidothalamic tractotomy (PTT)ablation, while the rest reported unilateral ablations (5, 9, 11, 20, 22–26, 28, 30–36). Regarding the surgical targets: 11 studies (5, 9, 11, 22, 23, 30–32, 34–36) selected the VIM nucleus; PTT was chosen as the target in 4 studies (20, 21, 27, 29); whereas STN (24, 33) and GPI (25, 28) were each selected in 2 studies.

**Table 1:** Characteristics of included studies.

Author, year	Study design	Patients	Follow-up	Age (Mean, Sd)	Sex (Male: Female)	PD duration year	Ablation target	Unilateral/ Bilateral	Baseline MDS-UPDRSIII scores (On state)	Baseline MDS-UPDRSIII scores (Off state)	Baseline total CRST scores
Chen et al. (2023) (36)	Retrospective	3	1 month	60.7 ± 6.0	3:0	7.3 ± 4.1	PTT + VIM	Unilateral		37.0 ± 8.0	
Dahmani et al. (2023) (5)	Prospective	10	1 year	55 ± 7.29	8:2	4.92 ± 1.59	VIM	Unilateral	29.7 ± 8.6		
Wang et al. (2023) (35)	Prospective	9	1 year	64.67 ± 6.12	8:1	8.22 ± 7.19	VIM	Unilateral			45.89 ± 8.94
Saporito et al. (2023) (34)	Prospective	18	6 months	65.4 ± 11.4	/	7.8 ± 4.63	VIM	Unilateral	30.0 ± 13.7		35.79 ± 14.39
Martinez-Fernandez et al. (2023) (33)	Prospective	32	36 months	56 ± 10.1	22:10	6.8 ± 2.8	STN	Unilateral	24.7 ± 7.4	36.8 ± 7.4	
Yin et al. (2022) (32)	Prospective	9	1 year	64.7 ± 6.1	8:1	7(5.5,9.0)	VIM	Unilateral	26 ± 7.41	57.33 ± 7.74	20 ± 7.78
Golfrè Andreasi et al. (2022) (31)	Prospective	10	6 months	62.3 (60.2, 72.3)	8:2	3.8(2.4,4.5)	VIM	Unilateral	22.5 ± 8.15		
Stanziano et al. (2021) (30)	Prospective	15	3 months	64 ± 7	13:2	6.8 ± 6	VIM	Unilateral		7.2 ± 1.9	
Eisenberg et al. (2021) (28)	Prospective	20	1 year	56.4 ± 11.3	13:7	9.9 ± 6.4	GPI	Unilateral		20.0 ± 5.6	
Gallay et al. (2021) (29)	Prospective	10	1 year	63 ± 5	5:5	10.2 ± 10.6	PTT	Bilateral		41.0 ± 20.0	
Zur et al. (2020) (26)	Prospective	17	6 months	65 ± 8	13:4	6 ± 3	/	Unilateral	5.4 ± 1.6		
Gallay et al. (2020) (27)	Prospective	51	1 year	67.3 ± 10.1	37:14	10 ± 5.3	PTT	Unilateral/ Bilateral			
Jung et al. (2019) (25)	Prospective	8	6 months	59.8(52–73)	/	10.1(6–14)	GPI	Unilateral	8.5 ± 2.8	30.1 ± 6.2	
Martinez-Fernandez et al. (2018) (24)	Prospective	10	6 months	59.5 ± 10.1	6:4	6.3 ± 2.5	STN	Unilateral	21.5 ± 6.3	32.7 ± 5.4	
Zaaroor et al. (2018) (9)	Prospective	9	2 years	59.4 ± 8.4	8:1	5.3 ± 3.3	VIM	Unilateral	24.9 ± 8.0		
Iacopino et al. (2018) (23)	Prospective	4	3 months	68 ± 4.74	4:0	14 ± 11.3	VIM	Unilateral	36.5 ± 12.5		
Fasano et al. (2017) (22)	Retrospective	3	6 months	76.3 ± 4.0	3:0	10.3 ± 2.1	VIM	Unilateral	27.0 ± 1.0		
Wegener et al. (2016) (21)	Retrospective	3	6 months	61.1 ± 13.7	/	8.9 ± 5.1	PTT	Unilateral/ Bilateral	30.6 ± 10.5		
Schlesinger et al. (2015) (11)	Prospective	7	1 year	59.4 ± 9.8	6:1	5.4 ± 2.8	VIM	Unilateral			
Magara et al. (2014) (20)	Prospective	13	3 months	64.5 ± 12.8	8:5	9.7 ± 6.3	PTT	Unilateral	18.7 ± 7.2		

Pallidothalamic tractotomy (PTT), ventral intermediate nucleus (VIM), globus pallidus internus (GPI), subthalamic nucleus (STN). On state: on- medication states. Off state: off-medication states. Data are expressed as median (interquartile range) and mean ± standard deviation.

### 3.2 Tremor scores

#### 3.2.1 MDS-UPDRSIII scores (on-medication and off-medication states)

**Figure 2:**
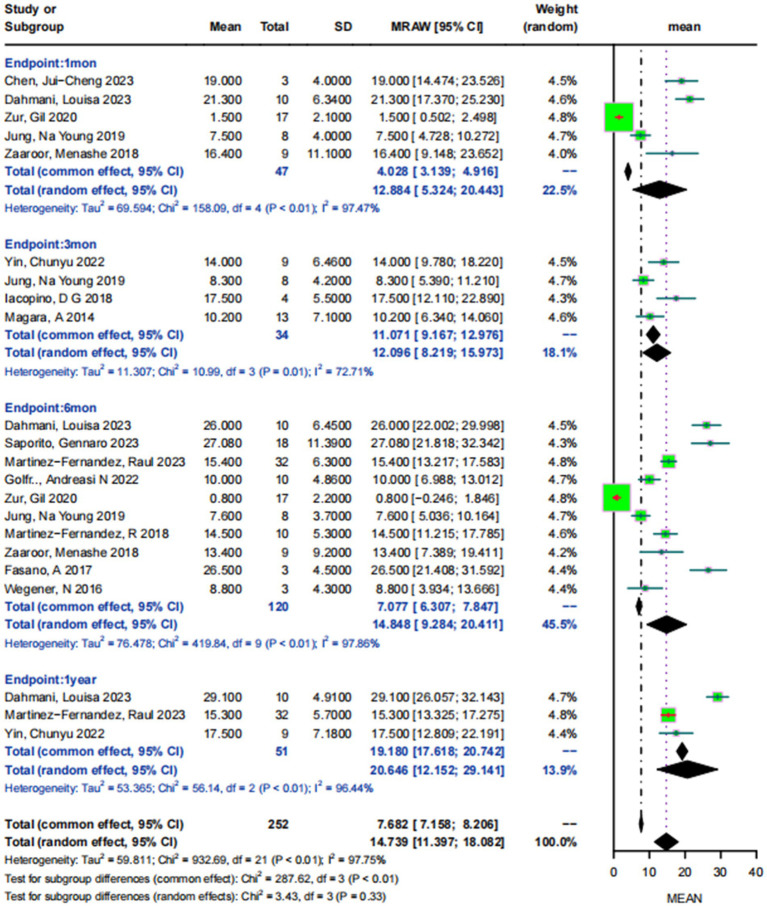
Forest plot of MDS-UPDRSIII scores at 1 month, 3 months, 6 months, and 1 year post-MRgFUS treatment in the on- medication states.

The mean MDS-UPDRSIII score for drug-resistant PD patients in the on-medication states on the treatment side at baseline was 27.77 ± 13.03. Five studies (5, 9, 25, 26, 36) involving 47 patients reported the mean MDS-UPDRSIII scores at 1 month from baseline to non-pharmacological status, which showed that the scores showed a high degree of heterogeneity (*I*^2^ = 96.9%, *p* < 0.05), and the pooled standard mean difference was 12.88 (95% CI:5.32–20.44).Four studies (20, 23, 25, 32) involving 34 patients reported mean MDS-UPDRS III scores at 3-month postoperative follow-up, showing a high degree of heterogeneity (I^2^ = 72.71%, *p* < 0.05), with a combined score of 12.10 (95% CI: 8.22–15.97). Ten studies (5, 9, 21, 22, 24–26, 31, 33, 34) involving 120 patients reported mean MDS-UPDRSIII scores at 6-month postoperative follow-up, showing a high degree of heterogeneity (*I*^2^ = 97.86%, *p* < 0.05), with a combined score of 14.85 (95% CI: 9.28–20.41). Three studies (5, 32, 33) concerning 51 patients and reporting the mean MDS-UPDRSIII scores at 1-year postoperative follow-up showed a high level of heterogeneity (*I*^2^ = 96.44%, *p* < 0.05), with a pooled result of 20.65 (95% CI: 12.15–29.14) (Figure 2).

**Figure 3:**
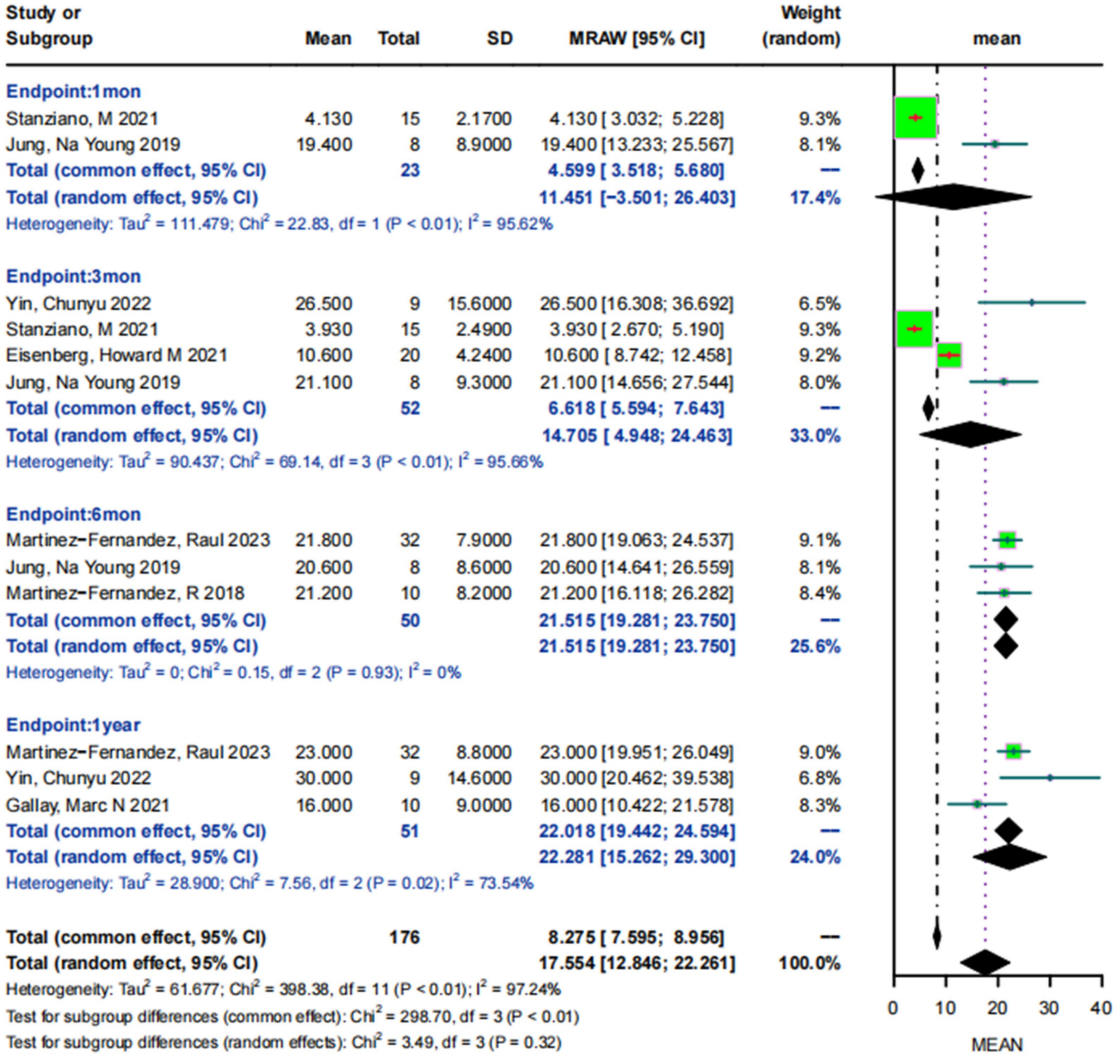
Forest plot of MDS-UPDRSIII scores at 1 month, 3 months, 6 months, and 1 year post-MRgFUS treatment in the off-medication states.

Mean MDS-UPDRSIII scores for patients with drug-resistant PD who were off-medication states on the treatment side at baseline were 31.65 ± 12.75. The 2 studies (25, 30) involving 23 patients reported the mean MDS-UPDRSIII scores at 1 month postoperatively, and their results showed a high degree of heterogeneity in the scores (*I*^2^ = 95.62%, *p* < 0.05), with a combined analysis of 11.45 95% CI:−3.50–26.40. Four studies (25, 28, 30, 32), involving 52 patients, reported mean MDS-UPDRSIII scores at 3-month postoperative follow-up exhibited a high degree of heterogeneity (*I*^2^ = 95.66%, *p* < 0.05), and 14.71 (95% CI:4.95–24.46) after combining. Three studies (24, 25, 33) involving 50 patients reported the mean MDS-UPDRSIII score at 6-month postoperative follow-up, showing a heterogeneity of scores of 0 (*I*^2^ = 0, *p* < 0.05) and a combined MDS-UPDRSIII score of 21.52 (95% CI:19.28–23.75). Three studies (29, 32, 33) involving 51 patients reported the mean MDS-UPDRSIII scores at 1-year postoperative follow-up, showing a high degree of heterogeneity (*I*^2^ = 73.54%, *p* < 0.05), with a combined result of 22.28 (95% CI: 15.26–29.30) (Figure 3).

In both states, MRgFUS treatment effectively reduced the MDS-UPDRSIII scores, indicating its efficacy. Comparatively, the baseline score in the “off” medication state was higher, but the post-treatment reduction trend mirrored the “on” state, suggesting a potentially more pronounced effect in the “off” state. This could be attributed to the higher baseline score in the “off” state, offering more room for improvement. Over time, the therapeutic effect diminishes, possibly due to the progressive nature of the disease.

As can be seen from the Table 2, MRgFUS had a positive impact on the treatment of motor symptoms in drug-resistant PD patients, especially in terms of tremor and bradykinesia. It is important to note, however, that the treatment effect was relatively small in stiffness symptoms. These results emphasize the potential of MRgFUS treatment in improving different motor symptoms in patients with Parkinson’s disease, but also highlight the variability between different symptoms.

**Table 2:** Detailed changes in specific sections of MDS-UPDRSIII.

Author, year	Locomotor condition		Baseline	3-month follow-up	6-month follow-up	1-year follow-up
Martinez-Fernandez et al. (2023) (33)	Tremor	OFF	5.2 + 2.3		1.2 + 1.4	1.1 + 1.6
		ON	3.7 + 1.9		0.9 + 1.3	0.5 + 1.0
	Bradykinesia	OFF	10.3 + 2.5		5.0 + 2.8	5.4 + 3.0
		ON	7.3 + 2.4		3.6 + 2.8	3.9 + 2.6
	Rigidity	OFF	3.5 + 0.9		1.5 + 1.3	1.7 + 1.2
		ON	2.8 + 1.1		0.9 + 1.0	1.1 + 1.2
Yin et al. (2022) (32)	Tremor	OFF	19.0 (14.5, 21.0)	8.0 (5.0, 10.5)		7.0 (4.0, 12.5)
		ON	6.0 (1.5, 11.0)	2.0 (0.0, 2.5)		0.0 (0.0, 2.5)
	Bradykinesia	OFF	23.0 (16.5, 25.0)	16.0 (9.5, 19.5)		17.0 (10.0, 23.5)
		ON	8.0 (6.5, 12.0)	6.0 (4.5, 10.5)		9.0 (5.5, 10.5)
	Rigidity	OFF	9.0 (7.0, 11.0)	8.0 (7.0, 11.0)		9.0 (7.0, 11.5)
		ON	6.0 (5.5, 6.5)	6.0 (5.5, 8.5)		7.0 (6.0.10.0)
Golfrè Andreasi et al. (2022) (31)	Tremor	ON	8.0 (7.0; 9.8)		3.0 (1.5; 4.8)	
	Bradykinesia	ON	6.5 (4.5; 8.75)		6.0 (3.0; 6.8)	
	Rigidity	ON	2.0 (2.0; 3.0)		0.5 (0.0; 2.0)	
Gallay et al. (2021) (29)	Tremor	OFF	13 ± 6			0.9 ± 2.1
		ON	11 ± 6			–
	Bradykinesia	OFF	14.0 ± 7.7			5.8 ± 4.5
		ON	12.6 ± 6.9			–
	Rigidity	OFF	6.4 ± 3.8			1.8 ± 1.8
		ON	5.3 ± 3.2			–
Martinez-Fernandez et al. (2018) (24)	Tremor	OFF	4.2 ± 2.1		1.2 ± 1.8	
		ON	3.7 ± 1.9		0.9 ± 1.7	
	Bradykinesia	OFF	9.4 ± 2.7		5.6 ± 2.9	
		ON	6.5 ± 2.0		4.7 ± 2.1	
	Rigidity	OFF	2.9 ± 0.7		0.8 ± 0.8	
		ON	2.2 ± 1.2		5.8 ± 3.5	

OFF, off-medication states; ON, on- medication states. Data are expressed as median (interquartile range) and mean ± standard deviation.

### 3.3 CRST scores

In three studies, total CRST scores were documented. Wang et al. (35) observed an initial score of 45.89 ± 8.94, which significantly reduced to 17.89 ± 11.92 1 year post-surgery. Saporito et al. (34) recorded a baseline score of 35.79 ± 14.39, which dropped to 23.03 ± 10.95 6 months postoperatively. Similarly, Yin et al. (32) registered an initial score of 20 ± 7.78, declining to 3.44 ± 2.83 1 year post-intervention. Due to the insufficiency of data, a meta-analysis on the aforementioned results could not be conducted.

### 3.4 Recurrent events of tremor

Eisenberg et al. (28) reported a recurrence of tremor in a PD patient at month 3 after GPI-targeted surgery. Zaaroor et al. (9) mentioned 2 patients experiencing tremor recurrence, one with significant recurrence within 3 months of undergoing the VIM procedure and the other with minor recurrence within 6 months. Schlesinger et al. (11) documented that 1 patient each experienced transient mild tremor recurrence at various time points after VIM surgery, including 1 week, 1 month, and 6 months. Magara et al. (20) noted that four patients experienced tremor recurrence within 3 months of PTT surgery.

### 3.5 Adverse events

We have summarized the adverse events during and after surgery in the included studies (Table 3). Generally, the procedure was safe for these patients, with the majority of adverse events being mild and transient.

**Table 3:** Summary of adverse events during and after the procedure.

Author, year	Adverse events during the procedure	Adverse events after the procedure
Chen et al. (2023) (36)	Headache (*n* = 1), dizziness/vertigo (*n* = 2), head pain/heat sensation (*n* = 1), not persistent at the follow-up.	0
Dahmani et al. (2023) (5)	0	At 6 months: target hand’s inflexible movement and slow reaction (*n* = 1), slight shaking in the treated leg (*n* = 1). By 12 months, all adverse effects resolved. Other complications were discussed with the conditions of ET patients.
Wang et al. (2023) (34)	0	Mild dizziness (*n* = 4), which was relieved within 24 h.
Saporito et al. (2023) (35)	#	#
Martinez-Fernandez et al. (2023) (33)	0	4–6 months post-treatment AE included dyskinesias (*n* = 3), clumsiness/weakness (*n* = 1), facial asymmetry (*n* = 1), dysarthria (*n* = 2), reduced verbal fluency (*n* = 1), unsteady gait (*n* = 1), weight gain (*n* = 3). Most were mild. At 3 years, issues were reduced verbal fluency (*n* = 1), mild dysarthria (*n* = 1), and clumsy hand (*n* = 1).
Yin et al. (2022) (32)	Headache (*n* = 1) and dizziness (*n* = 2), which disappeared after the operation was completed.	Post-operation, patients reported gait disturbance (*n* = 3), tongue tip numbness (*n* = 4), and hypogeusia (*n* = 1). Two had gait issues and one had tongue tip numbness resolve in a month. All other symptoms improved within 3–12 months. All responses were mild to moderate.
Golfrè Andreasi et al. (2022) (31)	No serious AEs (i.e., associated with new or prolonged hospitalization, permanent disability, or death) were found in either MRgFUS VIM thalamotomy.	
Stanziano et al. (2021) (30)	NA	NA
Eisenberg et al. (2021) (28)	Related to placement of the stereotactic frame (headache, facial edema) (*n* = 4), 17 of the AEs were transient, which included the only severe AEs (2 with transient sonication-related head pain, 1 with transient nausea and vomiting).	Nausea/vomiting and headache affected 3 patients each, while 7 had sonication-related head pain. Neurological AEs from the procedure: visual field deficit (1 mild, transient), dysarthria (*n* = 4; 2 mild, 2 moderate), cognitive disturbance (1 mild), fine motor deficit (2 mild), facial weakness (1 mild), balance difficulties (1 moderate). 20 AEs persisted: fine motor difficulties (1 mild), dysarthria (3; 1 mild, 2 moderate), balance difficulties (1 mild)
Gallay et al. (2021) (29)	Sonications were painful for a few seconds (*n* = 1).	Hiccup, breathing and speech issues (*n* = 1, regressed at 10 months); gait disturbance (*n* = 1, normalized at 3 months). At 1 year, uncontrollable laughter and blepharospasms (*n* = 1).
Zur et al. (2020) (26)	NA	NA
Gallay et al. (2020) (27)	Sonications were painful (*n* = 7, for a few seconds), scalp hypoesthesia (*n* = 1, recovered after 3 months).	Intense anxio-depressive episode (*n* = 1, relapsed after 1 year post-op). At 3 months: speech difficulties (*n* = 7), hiccup with breathing and speech issues (*n* = 1, persisted for months), gait disturbance (*n* = 1).
Jung et al. (2019) (25)	Mild headache (*n* = 8).	After frame removal, pin-site pain occurred (*n* = 8, typically no medication needed for pain). Back pain from fixed positioning (*n* = 4, alleviated with analgesics). Neurological issues, dysarthria, and grade-III right motor hemiparesis noted (*n* = 1, fully resolved in 2 days).
Martinez-Fernandez et al. (2018) (24)	Transient cranial warmth (*n* = 2), pin-site head pain (*n* = 6), nausea (*n* = 4), back pain (*n* = 2), anxiety (*n* = 2), and high blood pressure (*n* = 5).	Transient gait ataxia (*n* = 6) and facial palsy (*n* = 1, resolved during follow-up). Post-discharge behavioral changes like impulsivity (*n* = 2, resolved in a month). Off-drug choreic dyskinesias in shoulder/arm (*n* = 1, gone by 6 months) and involuntary movements in treated arm (*n* = 1). Subjective speech disturbance (*n* = 1). Weight gain (*n* = 2), fatigue (*n* = 1), and anxiety (*n* = 1).
Zaaroor et al. (2018) (9)	#	Gait ataxia (*n* = 1). Other complications were discussed alongside ET patient conditions.
Iacopino et al. (2018) (23)	#	#
Fasano et al. (2017) (22)	0	Transient local pain/burning (*n* = 2), dizziness (*n* = 1) and headache (*n* = 1), dysarthria (*n* = 1) and eyelid weakness (*n* = 1). Persistent numbness/paresthesia (*n* = 1) and hemiparesis (*n* = 1).
Wegener et al. (2016) (21)	0	Transient dysphagia (*n* = 1).
Schlesinger et al. (2015) (11)	Headache (*n* = 3), dizziness (*n* = 2), vertigo (*n* = 4), and lip paresthesia (*n* = 1, resolved after target was repositioned 1 mm anteriorly).	Hypogeusia (*n* = 1), subjective unsteady feeling when walking (*n* = 1, resolved), and disturbance when walking tandem (*n* = 1, resolved at 2-month follow-up).
Magara et al. (2014) (20)	0	0

Studies with no complications are labeled “0.” If no data on complications was given for a time period, it’s marked “NA.” #Adverse events for PD were grouped with other diseases, so exact PD numbers are unknown. Adverse event severity was categorized: mild (minimal impact), moderate (interferes with daily activities), or severe (prevents daily activities).

Adverse events from MRgFUS can be primarily categorized into two main types: neurological complications and side effects associated with MRI/ultrasound or the frame. Neurological complications can be further delineated into: sensory deficits (e.g., taste disturbances, sensory loss, visual field defects, paresthesia, numbness, or burning sensations, total of 20 cases), motor disturbances (e.g., facial or limb weakness, eyelid spasms, total of 12 cases), ataxia (e.g., unsteady gait, hand coordination difficulties, total of 18 cases), speech disorder (total of 18 cases), cognitive and emotional disturbances (e.g., anxiety, depression, fatigue, and behavioral changes, total of 10 cases), hypertension (total of 5 cases), and thalamotomy-related dizziness (*n* = 5) and headache (*n* = 4). Side effects related to MRI/ultrasound or the frame primarily included: headache (30 cases), dizziness (10 cases), head burning sensation (3 cases), facial swelling (4 cases), nausea and vomiting (total of 4 cases), pain induced by ultrasound (8 cases), and back pain (6 cases). Additionally, the studies reported instances of hiccupping, respiratory difficulties (2 cases), weight gain (5 cases), and swallowing difficulties (1 case).

Out of the total, 65 patients (representing 25.2%) experienced side effects associated with MRI/ultrasound or the frame, with headache and dizziness being the most common. These events usually subsided on their own within a few days without the need for specialized intervention. The most commonly reported neurological adverse events were sensory abnormalities, ataxia, and speech disorders, which generally improved within 3 months post-operation and had a minimal impact on patients’ daily lives. The severity of most adverse reactions ranged from mild to moderate. The only three severe adverse events reported were by Eisenberg et al. (28), which included two cases of transient headache related to ultrasound and one case of transient nausea and vomiting; neither of these met the United States Food and Drug Administration (FDA) definition for severe adverse reactions. In a study by Gallay et al. (29), one patient experienced uncontrollable laughter and eyelid spasms a year post-operation, and in another of their studies (27), a patient underwent a brief yet intense episode of anxiety and depression, which then recurred after more than a year post-operation. Fasano et al. (22) reported persistent side effects in two patients: numbness and hemiparesis accompanied by hemihypoesthesia. It remains uncertain whether these persistent adverse events will fade with extended follow-up.

### 3.6 Quality of the evidence

Two researchers independently evaluated the studies using the ROBINS-I scale (37). The included studies were assessed for potential biases in seven areas: confounding bias, selection of participants bias, intervention classification bias, intention to intervene deviation bias, missing data bias, outcome measurement bias, and selective reporting bias. These evaluations are presented in Table 4. In cases of disagreement, the issues were resolved through mutual consultation or determined through a discussion with a third party.

**Table 4:** Robins-I quality rating scale.

Author, year	Confounding bias	Selection bias	Intervention classification bias	Intention-to-intervention bias	Missing data bias	Outcome measurement bias	Selective reporting bias	Overall risk of bias
Chen et al. (2023) (34)	2	1	2	1	1	2	1	2
Dahmani et al. (2023) (5)	3	2	1	2	1	2	1	3
Wang et al. (2023) (33)	3	2	1	2	1	2	1	3
Saporito et al. (2023) (32)	4	2	1	1	1	1	2	4
Martinez-Fernandez et al. (2023) (31)	1	2	5	1	1	1	1	1
Yin et al. (2022) (30)	3	2	1	1	1	2	1	2
Golfrè Andreasi et al. (2022) (29)	2	1	1	5	1	2	1	2
Stanziano et al. (2021) (28)	2	5	1	1	2	2	2	2
Eisenberg et al. (2021) (26)	2	1	1	1	1	2	1	2
Gallay et al. (2021) (27)	3	1	1	1	1	2	2	2
Zur et al. (2020) (24)	2	2	1	1	1	2	1	2
Gallay et al. (2020) (25)	2	1	1	1	2	2	2	2
Jung et al. (2019) (9)	2	1	1	1	1	2	2	2
Martinez-Fernandez et al. (2018) (23)	3	2	2	1	1	2	1	3
Zaaroor et al. (2018) (9)	4	2	1	1	1	2	1	2
Iacopino et al. (2018) (22)	3	2	1	2	1	2	1	3
Fasano et al. (2017) (21)	3	2	1	1	1	1	2	3
Wegener et al. (2016) (11)	3	2	1	1	2	2	1	3
Schlesinger et al. (2015) (11)	3	1	1	1	2	2	1	3
Magara et al. (2014) (20)	3	2	1	1	1	1	1	3

Low, 1; Moderate, 2; Serious, 3; Critical, 4; NI, 5.

Of the 20 studies selected. A few studies had some quality issues, including potential confounders and selective reporting of risk. However, some studies performed relatively well in certain aspects, such as lower risk bias and better methodological quality. Overall, these studies provide preliminary information about MRgFUS treatment for drug-resistant PD-related tremor, but caution is needed in interpreting the results, especially in the presence of potential wind traps. Future studies should focus more on methodologic quality to further validate the efficacy and safety of this treatment.

## 4 Discussion

MRgFUS as a novel non-invasive intervention technique has gradually become a new option for treating medication-resistant PD patients. To our knowledge, this is the first comprehensive meta-analysis of the efficacy of MRgFUS in the treatment of PD. Overall, this study suggests that MRgFUS treatment for drug-resistant PD is both effective and safe.

In both states, MRgFUS significantly reduced the MDS-UPDRSIII scores. However, as time post-surgery progresses, scores tend to rise, suggesting a potential diminishing therapeutic effect, warranting further longitudinal studies. We recognize this and also consider that Parkinson’s disease is a progressive neurodegenerative disease. The progression of this disease may be an important reason for the rise in scores. Given this, MRgFUS’s capability for repeated treatments emerges as a distinct advantage. Since MRgFUS primarily targets symptoms unresponsive to medication, improvements during the drug-off state are particularly noteworthy for an accurate assessment of the surgical intervention’s benefits. Thus, notable symptom relief by MRgFUS during the off-medication states, given its critical role in patients’ daily challenges, represents a crucial therapeutic milestone. Nonetheless, enhancements in the on-medication states also epitomize the overall treatment efficacy. In line with previous reports, our data indicates that speech disorder, ataxia, and sensory abnormalities are the most common adverse events in the neurological system after MRgFUS treatment (16). Complications related to MRI/ultrasound or the frame are typically transient reactions during the treatment process, such as headaches, dizziness, nausea, vomiting, scalp numbness, or a burning sensation. Our findings reveal that over a quarter of patients experienced ultrasound-related complications, with headaches and dizziness being the most frequent. Additionally, the use of anesthetics is avoided during the MRgFUS ultrasound procedure, offering a safer treatment alternative for patients at high risk from general anesthesia. Compared to other therapeutic technologies, an advantage of MRgFUS is that most surgery-related complications can be detected in real-time during surgery. This allows physicians to mitigate or reverse most side effects by adjusting the initial treatment target.

A total of 4 different surgical targets were used in the 20 studies we reviewed, with VIM being the most commonly used surgical target. MRgFUS produces varying effects and potential complications across different targets. VIM therapy is commonly used to suppress tremor symptoms, but may be accompanied by sensory or motor impairment, resulting in sensory abnormalities, muscle weakness, or dyskinesia. Some patients may also experience pain after the procedure, which may require additional management. STN treatment has an ameliorating effect on major motor symptoms, such as rigidity, tremor, and bradykinesia, and also reduces the dose of levodopa-related treatments. However, the treatment may also lead to movement disorders and speech or cognitive problems. GPI treatment provides significant relief from almost all symptoms of drug-resistant PD, especially when accompanied by cognitive decline and mood disorders, but may also trigger motor deficits and language or cognitive dysfunction. PTT treatment produces positive results in dyskinesia and dystonia, but may result in abnormal sensations or increased pain after the procedure, as well as some temporary headaches or discomfort (38). It is important to note that complications of the MRgFUS procedure can vary greatly from patient to patient, and with improvements in surgical techniques, it has become possible to reduce the risk of complications. Future research should be directed toward exploring which target delivers the best results in MRgFUS therapy and whether there are adverse effects associated with target selection. In addition, MRgFUS single-target thalamotomy has not demonstrated significant efficacy in some of the motor symptoms of PD such as rigidity, bradykinesia, and gait disturbances, as well as in a number of non-motor symptoms such as cognitive deficits, affective problems, and sleep disorders. However, Chen et al. (36) have demonstrated that dual-targeted MRgFUS significantly reduced resting and locomotor tremor in drug-resistant PD. Future research directions should focus on exploring the potential benefits of MRgFUS for drug-resistant PD patients in terms of non-motor symptoms in order to improve the overall quality of life of patients.

Moreover, numerous studies have concurrently addressed the efficacy and safety of MRgFUS in treating both ET and drug-resistant PD. This conflation precluded their inclusion in our analysis, potentially affecting the comprehensiveness of our data. Given this backdrop, we advocate for more dedicated clinical studies focusing solely on drug-resistant PD, especially since the safety and efficacy of MRgFUS in treating ET have already been established.

We concluded that selection of appropriate patients for MRgFUS treatment is critical to ensuring the efficacy and safety of the treatment. Current selection criteria may be based primarily on patient history, disease stage, and ancillary tests. However, as our understanding of PD grows, there may be other biomarkers or neuropsychological assessment tools that can more accurately predict which patients are most likely to benefit from MRgFUS therapy. Considering that the duration of PD and lesions vary widely from patient to patient, a uniform treatment approach may not be appropriate for all patients. Therefore, new metrics with predictive value could help to individualize treatment. It is recommended to consider combining MRgFUS with other non-pharmacological treatments (e.g., cognitive rehabilitation, physical therapy, or DBS) to assess whether treatment effects can be further enhanced. In exploring this direction, we should focus on improving the overall quality of life of patients and advancing individualized treatment to ensure the best outcome for each patient.

### 4.1 Limitations

This study primarily relies on the MDS-UPDRSIII for assessing treatment outcomes. While it is a key tool for evaluating PD, the inclusion of other crucial indicators such as quality of life and mental state was limited by data availability, potentially hindering a comprehensive understanding of the MRgFUS treatment effects. In addition, most of our studies had limited sample sizes and short follow-up periods.

## 5 Conclusion

MRgFUS is a potential option for the treatment of drug-resistant PD-related tremor with satisfactory efficacy and safety. Speech disorders, ataxia and sensory abnormalities are the most common postoperative side effects, but the symptoms are mild and usually transient. However, because MRgFUS is a relatively new technique, follow-up data and randomized clinical trials are quite limited. More rigorous study designs, larger sample sizes, and longer follow-up times are needed in the future to further investigate the efficacy, safety, and durability of MRgFUS in the treatment of drug-resistant PD-related tremor in order to determine its long-term benefits in the management of drug-resistant PD-related tremor.

## Data availability statement

The original contributions presented in the study are included in the article/supplementary material, further inquiries can be directed to the corresponding author.

## Author contributions

XT: Conceptualization, Writing – original draft. RH: Formal analysis, Software, Supervision, Writing – review & editing. PH: Formal analysis, Writing – review & editing. JY: Conceptualization, Writing – review & editing.
